# Use of 4 Open-Ended Text Responses to Help Identify People at Risk of Gaming Disorder: Preregistered Development and Usability Study Using Natural Language Processing

**DOI:** 10.2196/56663

**Published:** 2024-12-31

**Authors:** Paweł Strojny, Ksawery Kapela, Natalia Lipp, Sverker Sikström

**Affiliations:** 1 Faculty of Management and Social Communication Jagiellonian University Krakow Poland; 2 Sano Centre for Computational Medicine International Research Foundation Kraków Poland; 3 Department of Psychology Lund University Lund Sweden

**Keywords:** gaming disorder, natural language processing, machine learning, mental health, NLP, text, open-ended, response, risk, psychological, Question-based Computational Language Assessment, QCLA, transformers-based, language model analysis, Polish, Pearson, correlation, Python

## Abstract

**Background:**

Words are a natural way to describe mental states in humans, while numerical values are a convenient and effective way to carry out quantitative psychological research. With the growing interest of researchers in gaming disorder, the number of screening tools is growing. However, they all require self-quantification of mental states. The rapid development of natural language processing creates an opportunity to supplement traditional rating scales with a question-based computational language assessment approach that gives a deeper understanding of the studied phenomenon without losing the rigor of quantitative data analysis.

**Objective:**

The aim of the study was to investigate whether transformer-based language model analysis of text responses from active gamers is a potential supplement to traditional rating scales. We compared a tool consisting of 4 open-ended questions formulated based on the clinician's intuition (not directly related to any existing rating scales for measuring gaming disorders) with the results of one of the commonly used rating scales.

**Methods:**

Participants recruited using an online panel were asked to answer the Word-Based Gaming Disorder Test, consisting of 4 open-ended questions about gaming. Subsequently, they completed a closed-ended Gaming Disorders Test based on a numerical scale. Of the initial 522 responses collected, we removed a total of 105 due to 1 of 3 criteria (suspiciously low survey completion time, providing nonrelevant or incomplete responses). Final analyses were conducted on the responses of 417 participants. The responses to the open-ended questions were vectorized using HerBERT, a large language model based on Google's Bidirectional Encoder Representations from Transformers (BERT). Last, a machine learning model, specifically ridge regression, was used to predict the scores of the Gaming Disorder Test based on the features of the vectorized open-ended responses.

**Results:**

The Pearson correlation between the observable scores from the Gaming Disorder test and the predictions made by the model was 0.476 when using the answers of the 4 respondents as features. When using only 1 of the 4 text responses, the correlation ranged from 0.274 to 0.406.

**Conclusions:**

Short open responses analyzed using natural language processing can contribute to a deeper understanding of gaming disorder at no additional cost in time. The obtained results confirmed 2 of 3 preregistered hypotheses. The written statements analyzed using the results of the model correlated with the rating scale. Furthermore, the inclusion in the model of data from more responses that take into account different perspectives on gaming improved the performance of the model. However, there is room for improvement, especially in terms of supplementing the questions with content that corresponds more directly to the definition of gaming disorder.

**Trial Registration:**

OSF Registries osf.io/957nz; https://osf.io/957nz

## Introduction

### Rating Scale Method Domination in Gaming Disorder Screening

Over the last decade, research on gaming disorder has been intensively conducted. More than 30 different rating scale measures have been suggested (see [1] for details), and new scales are under development (see, eg, [2]). Gaming disorder has recently been defined in the International Classification of Diseases (ICD)-11 diagnostic manual as a pattern of persistent or recurrent gaming behavior manifested by impaired control, increasing priority, and continued use despite occurrence of negative consequences [3]. This new definition is the result of progress in understanding the concept of gaming disorder, as a result of both gradually collected empirical data and a heated debate around the concept of gaming disorder [4]. Newer tools differ significantly from the earlier ones, as they correspond to the World Health Organization (WHO) diagnostic criteria (see [2] for more details). As the definition and methods of assessment for gaming disorder have been debated, the current aim was to present a new language-based approach that complements the existing rating scales methods.

The natural way to communicate subjective states to another person is to use words. However, for decades in the social sciences, this response format has been replaced by instructions requiring parameterization of one's state with numbers. For example, the overwhelming majority of screening measures for gaming disorder are based on closed questions with numerical responses. King et al [1] reviewed 32 measures of gaming disorder based on predefined test items, of which 23 used multiple response scales and 9 used binary responses (yes/no). Therefore, none of the gaming disorder measures allow the respondents to freely express their mental states.

The rating scale approach has both advantages and disadvantages. Undoubtedly, the main advantage of such methods is that they save time while maintaining satisfactory accuracy and reliability of the measurements. Assessment by professional clinicians is, of course, an alternative method; however, it has drawbacks as it is expensive, laborious, and subjective. Even if a complete clinical diagnosis yields more reliable results [5], it involves greater demands on both the participant and researcher, which has resulted in social scientists, for decades, frequently preferring evaluation based on rating scales.

However, limiting research to rating scale data also has obvious disadvantages; the most important limitations include the unnatural necessity to quantify mental phenomena or the susceptibility of the results to manipulation. The others are of a general nature and have been presented before (see, eg, [6]). Tausczik and Pennebaker [7] rightly noted that “Language is the most common and reliable way for people to translate their internal thoughts and emotions into a form that others can understand. The words and language, then, are the very stuff of psychology and communication.” As such, language fosters a precise and clear expression of desires, motives, perceived consequences, and the true intensity and nature of the gamer’s behavior. This provides motivation to exploit words as a more natural medium of communication during gaming disorder screening.

Another limitation during the assessment of gaming disorder is that rating scales are easy to manipulate. In their case, there is no room to mask the intention of the question. A person motivated to distort the assessment only has to underestimate or overestimate the reported numbers accordingly. Such actions can be reinforced by the desire to mislead others (eg, social desirability bias; [8]) or the need to maintain high self-esteem (eg, self-enhancement; [9]), which can play a significant role in the case of gaming disorder. In fact, a very high percentage (44%) of people with clinically diagnosed gaming disorder give false negative results in numerical self-reports [5]. Providing false information in the form of text is also possible but requires more effort than simply ticking a lower number on the questionnaire. In addition, natural language processing (NLP) with a sufficiently large database can be used to detect lying [10].

In summary, supplementing traditional screening methods with the analysis of open-ended responses seems like a necessary step to consider in the future, as describing mental states with words is more natural, potentially carries more information, and, in the future, may turn out to be resistant to manipulation.

### Natural Language Processing as a Potential Complement

Recent progress in NLP and artificial intelligence provides unprecedented opportunities to analyze text data. Such an analysis allows the respondent to freely choose words to describe their mental state. Previous research has demonstrated the effectiveness of this approach in the assessment of other areas of mental health. The estimates made by artificial intelligence on the basis of descriptive words provided results that were highly consistent with questionnaires that examined the same constructs. For example, in a series of 7 studies, the Pearson correlation coefficients between estimates based on open responses (processed using the context-free latent semantic analysis model) and the actual scores for life satisfaction and harmony on life scales ranged between *r*=0.47 and *r*=0.72 [11]. Importantly, the precision of the estimates turned out to be a function of the sample size (N=64), which was sufficient to obtain statistically significant results; however, with an increasing number of respondents, the trained algorithm turned out to be more effective.

Even better results were obtained by using relatively new transformer-based language representation models, such as Bidirectional Encoder Representations from Transformers (BERT [6]). The state-of-the-art precision comes from its transformer-based architecture, which uses self-attention to assign varying levels of importance to different components of input data such as natural language [12]. Unlike earlier language models, BERT is bidirectional, since it considers both preceding and following words while processing contextual information. The significance of such an approach can be illustrated with the word “flies,” which varies in meaning in different contexts (eg, “time flies” and “flies are annoying”); it is the accompanying words that determine how “flies” should be understood. Implementing BERT has improved the accuracy of estimates by 11% to 13% in the context of well-being [6].

To our knowledge, NLP, including transformer-based language representation models, has not previously been applied as a method to support and extend the screening for the risk of behavioral addictions. Particularly great potential lies in the sensitivity of recent transformer-based models, which may prove particularly important in the case of emotionally ambivalent and cognitively incoherent states reported by participants at risk of gaming disorder. Using NLP for gaming disorder diagnosis may be considered innovative and creative as it pioneers a transformative approach to mental health assessment. Traditional diagnostic methods often rely on self-reported questionnaires and clinical interviews, which can be subjective and limited in scope. By integrating NLP, we tap into a vast and underutilized resource: the natural, spontaneous language used by individuals in various digital contexts. This allows the identification of linguistic patterns and emotional signals that may be overlooked in conventional assessments. Furthermore, the idea of adopting NLP in the diagnosis of gaming disorder leverages the power of machine learning to process and analyze large data sets, uncovering insights with unprecedented depth and precision. Therefore, we decided to adapt the transformer-based model to assess gaming disorder. Our model was validated using the correlation of model estimates with the results of a traditional screening test.

### Hypotheses

We hypothesized that language-based assessments correlate significantly with rating scales (H1), the combined use of multiple open responses improves the accuracy of predicting gaming disorder compared with single open-ended questions (H2), and, in line with the findings in [6], that language-based assessments of gaming disorder will be as accurate as gaming disorder scale test-retest (H3).

## Methods

### Recruitment

Participants were recruited from the Polish research portal Ariadna [13] in November 2022.

A total of 522 individuals completed the questionnaire. After removing answers for reasons explained in the “Data Preparation and Cutoffs” section, the sample size was 417. All participants responded positively to the screening question: “have you played video games in the last 2 weeks (on your phone, computer, tablet or any electronic device)?”

### Open Practices

According to best practices, the data, code, and materials [14], as well as the preregistration that was time-stamped for the study protocol [15], are available for download on the Open Science Framework. Please note that we preregistered 2 additional hypotheses regarding solely mental health and well-being; the results are not reported here for the sake of brevity.

**Ethical Considerations**

The research was conducted following the laws and ethical principles for research and adhered to data protection under the General Data Protection Regulation (GDPR). The study was carried out in accordance with the Declaration of Helsinki and was approved by the Institutional Research Ethics Committee of Jagiellonian University (opinion number 133/2022). All of the participants gave informed consent before the study began. All of the study data were collected anonymously. We dedicated Zl 8 (US $2) as compensation for each person surveyed, which was given to them as part of a rewards program run by the data provider.

### Instruments and Methods

#### Gaming Disorder Self-Report

The Polish version of the Gaming Disorder Test (GDT, [16]; for the Polish version, see Multimedia Appendix 1) was used to validate the word-based measure. GDT is a questionnaire that contains 4 items rated on a 5-point Likert scale (ranging from 1: never to 5: very often). The Cronbach alpha in our sample was .898, which can be considered good, close to excellent internal consistency. The English version of the GDT is shown in Textbox 1.

**English version of the Gaming Disorder Test.**

1. I have had difficulty controlling my gaming activity.
2. I have given increasing priority to playing games over other interests and daily activities.
3. I have continued gaming despite the occurrence of negative consequences.
4. I have experienced significant problems in life (eg, personal, family, social, education, occupational) due to the severity of my gaming behavior.

#### Word-Based Gaming Disorder Test (WBGD-4)

We created the Word-Based Gaming Disorder Test (WBGD-4) consisting of 4 open-ended questions on which to base NLP predictions. The open-ended gaming-related questions were designed to mimic a conversation that would be held during a clinical diagnosis by a clinician. Therefore, all questions were proposed by a clinician with knowledge of gaming disorder. We deliberately did not interfere with the clinician suggestions and avoided revealing the purpose of the study and the tool that would be used as a reference point to determine the validity (ie, GDT).

In addition to the WBGD-4 questions, we added 2 questions to specifically measure and validate hypotheses related to well-being rating scales, which are not included in this article, as they are not the main focus of this study. For details, see the preregistration.

The WBGD-4 questions had to be answered with a word, phrase, or short sentence. There were 10 small text fields under each question, and filling at least 5 of them was mandatory. The short answer format was chosen based on previous studies that demonstrated higher predictive accuracy for short responses (ie, words) compared with completely free-text formats. The requirement of at least 5 short responses was motivated by previous data that indicated that predictive accuracy does not increase with more response alternatives [11]. Questions were asked and answered in Polish. The WBGD-4 questions were: (1) How does playing affect your life? (2) How does playing affect your emotions and thoughts when you are NOT playing? (3) What needs do your activity related to games satisfy? (4) How do you loved ones react to your playing?

#### Additional Measures

The 2 additional open-ended questions were related to mental health and the extent to which the respondent was able to achieve his life goals. The first was supposed to be answered with a few phrases, and the latter was meant to be answered with 5 to 10 words, phrases, or short sentences.

1. Describe whether and to what extent you can achieve your life goals. If not, what is preventing you from doing so?
2. Describe your mental health.

The severity of depression was assessed with the Polish version of the 9-item Patient Health Questionnaire (PHQ-9; [17]). This questionnaire is intended to detect depression in the initial psychological diagnosis. The PHQ-9 assesses the severity of depression using 9 items on a 4-point rating scale ranging from 0 (Not at all) to 3 (Nearly every day). The Polish version used in the study was created by the Mapi Research Institute [18] (for the Polish version, see Multimedia Appendix 2).

Generalized anxiety was measured using the 7-item Generalized Anxiety Disorder questionnaire [19], which has a 4-point scale ranging from 0 (Not at all) to 3 (Nearly every day). The Polish version used in the study was created by Mapi Research Institute [18] (for the Polish version, see Multimedia Appendix 3).

Well-being was measured using 2 scales, both in Polish: Harmony in Life Scale [20] and Satisfaction with Life Scale [21,22] (for the Polish version, see Multimedia Appendix 4). Both scales were used in the short 3-item form [11] using a 7-point rating scale ranging from 1 (Strongly disagree) to 7 (Strongly Agree). Additional measures are described in the preregistration and are not analyzed here.

The final original instrument in the study measured gaming involvement. This measure had 2 columns indicating the type of day (“weekday” or “weekend”) and 6 rows indicating the type of activity (such as “playing video games,” “thinking about video games,” “reading about video games,” “watching material about games,” “talking about games,” or “considering buying gaming-related content”). Participants were asked to fill out the table with the number of hours and minutes spent on each activity on a specific type of day. For the Polish version, see Multimedia Appendix 5.

### Procedure

After initial consent, sociodemographic information, such as age, gender, and educational level, was collected. In the next step, participants were asked to answer 6 open questions (WBGD-4 and 2 additional questions). Subsequently, they completed the rating scale measures in the following order: GDT, Satisfaction with Life Scale, Harmony in Life Scale, PHQ-9, 7-item Generalized Anxiety Disorder questionnaire, gaming involvement. All of the answers were collected during 1 online session.

### Statistical Analysis

The analysis was carried out using the Python programming language. The following libraries were implemented for computational purposes: Pandas [23], Numpy [24], Transformers [25], and Sklearn [26]. Moreover, the Matplotlib [27] library was used for data visualization.

#### Data Preparation and Cutoffs

Some participants used the same word or even the same sign as a placeholder in all required text fields, which also led to a very short questionnaire completion time (below 5 minutes, while the questionnaire took an average of 14 minutes to complete). Hence, we decided to expand our exclusion criteria beyond the preregistration guidelines, which relied on the algorithms of the Ariadna (data provider) software and manual deletion of irrelevant answers. Furthermore, sticking to the initial criterion of at least 5 words used to answer each question would have reduced our sample by 177. To avoid that, we decided to lower the requirements to at least 3 words.

Consequently, participants who answered the survey in 5 minutes or less were excluded, resulting in the removal of 20 participants. The responses to the text fields for each open question were then aggregated. Spelling errors and most punctuation errors were corrected manually. All repeated words were reduced to 1. We deleted 33 responses containing clearly irrelevant answers. Another 52 were excluded due to not meeting the word requirements, leaving us with a final sample of 417.

#### Feature Extraction

To extract numerical features for a predictive model, each text answer on the WBGD-4 was transformed into embeddings (ie, a numerical vector) using herBERT, a BERT-based model dedicated to the Polish language [28]. The last layer was used as the predictor in the model, as it showed the best performance on the data set.

The answers to each of the 4 open-ended WBGD-4 questions were transformed into 768 numerical representations. The embeddings of the 4 questions were then stacked together to create an array consisting of 3072 (4 × 768) numbers. Therefore, 5 feature sets were used in the analysis: 4 containing representations of answers to singular WBGD-4 questions and the last 1 containing representations of answers to each question stacked together.

#### Use of a Predictive Model

The ridge regression model was used to forecast the GDT scores on the basis of text embeddings. First, the data were randomized and partitioned using the k-fold cross-validation method with *k*=10 [29]. Each feature set was then assessed accordingly. This approach ensured that the data were effectively distributed across the different folds, allowing for robust evaluation and validation of the ridge regression model's performance.

#### Creation of Word Clouds

To identify which words most significantly drive the model’s predictions, we collected all words used in the answers to the individual questions then iteratively deleted each one from the text features. This approach measures changes in model performance in the absence of specific words. We calculated performance metrics for predictions with each word omitted using previously described methods and compared these with a baseline model in which no words were removed. The impact of each word was quantified using the *z* score of the change in performance, identifying words with significant *z* scores (*P*<.05) as key drivers of the predictions.

To visually illustrate the influence of specific words, we generated a word cloud, with word size proportional to the *z* score, indicating the extent of impact, and color coding based on *P* values to reflect statistical significance. Polish words were translated into English using the Google Translator application programming interface then manually adjusted.

## Results

### Demographic Information

The age of the final sample ranged from 15 years to 50 years (mean 33.9, SD 8.9 years). Of the 417 individuals, 228 (54.7%) identified as female, and 189 (45.3%) identified as male. None of the respondents chose the “other” option. As all participants were Polish, it is important to note that the sample was not representative of other nationalities. Detailed demographic data can be found in Table 1.

**Table 1:** Demographic data of the respondents (N=417).

Characteristics		Results, n (%)
**Gender**		
	Male	189 (45.3)
	Female	228 (54.7)
**Education level **		
	Primary	7 (1.7)
	Lower secondary education	10 (2.4)
	Vocational (without high school diploma)	22 (5.3)
	Incomplete secondary education	9 (2.2)
	General secondary education	46 (11)
	Vocational secondary education (with high school diploma)	74 (17.7)
	Incomplete higher education	9 (2.2)
	Postsecondary school (vocational)	37 (8.9)
	Higher, a bachelor's or engineering degree	50 (12)
	Higher, master’s degree	145 (34.8)
	Doctoral education	8 (1.9)

### GDT Distribution

**Figure 1:**
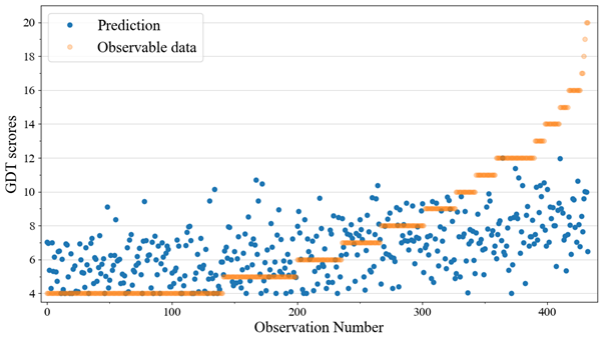
Observable Gaming Disorder Test (GDT) scores and predictions made by the model using all questions as its feature.

The results of the GDT were heavily skewed right (mean 7.4, SD 3.68), with only 2.4% (10/417) of the participants classified as suspected of having gaming disorder, as indicated by scores of 4 or 5 in each response. Almost one-third (131/417, 31%) achieved a minimal number of points, which means they marked “1” in every question (Figure 1); in the data shown in the figure, model predictions lower than 4 were increased to respond to the minimum attainable GDT score. Men (mean 7.94, SD 3.91) differed significantly from women (mean 6.96, SD 3.41; t_415_= 2.75, *P*=.007). The illustration can be found in Figure S1 in Multimedia Appendix 6.

### Text Responses

The text responses varied in length and content. The mean number of words and letters used (Table 2), as well as the words that appeared the most frequently (Table 3) are presented in the tables.

**Table 2:** Mean numbers of letters and words used in the answers to the 4 Word-Based Gaming Disorder Test (WBGD-4) questions.

Answers	Q1	Q2	Q3	Q4
Letters, mean (SD)	73.2 (27.09)	57.6 (21.16)	57.65 (15.84)	54.95 (17.67)
Words, mean (SD)	8.7 (3.74)	7.49 (3.3)	6.56 (2.05)	7.0 (2.68)

**Table 3:** Most frequently used words in the answers to each Word-Based Gaming Disorder Test (WBGD-4) question.

Words for each question		Times used, n
**Q1**		
	time	265
	relax	119
	entertainment	59
	fun	56
	relaxes	52
**Q2**		
	not	212
	lack	103
	affects	78
	oneself	57
	calmness	53
**Q3**		
	relax	135
	entertainment	73
	time	73
	fun	56
	joy	49
**Q4**		
	not	162
	oneself	57
	lack	52
	without	45
	anger	42

### Predictive Model Performance: Hypothesis Testing

We evaluated 4 ridge regression models based on responses from each WGBD-4 question separately and 1 model responding to a combination of responses from the 4 questions. To test H1, we calculated the correlations between the linguistic model estimates and the scores obtained using the self-report scale, and we also calculated the mean absolute error (MAE). The estimations of all 5 models correlated positively with the results obtained using the GDT. Question 1 had the lowest performance on both validation metrics, while Questions 4 and 2 had the most accurate predictions (Table 4). Using a combination of features from all the questions had a significantly higher correlation, compared with the most performing singular question (*z*=–2.33, *P*=.01 [30]). In terms of MAE, comparing the results of each model with the GDT results gave an average error of less than 3 (on a scale of 4 to 20); again, the combination of all 4 questions performed much better, giving an average error of 2.59.

**Table 4:** Ridge regression model performance on each feature set for the entire sample (general) and broken down by gender.

Features	General			Male			Female		
	*r*	*P* value	MAE^a^	*r*	*P* value	MAE	*r*	*P* value	MAE
Q1, Q2, Q3, Q4	0.476	<.001	2.59	0.527	<.001	2.74	0.427	<.001	2.47
Q2, Q3, Q4	0.475	<.001	2.62	0.487	<.001	2.83	0.429	<.001	2.47
Q2, Q4	0.456	<.001	2.65	0.473	<.001	2.86	0.419	<.001	2.49
Q4	0.406	<.001	2.75	0.436	<.001	2.96	0.341	<.001	2.6
Q2	0.369	<.001	2.77	0.362	<.001	3.03	0.353	<.001	2.57
Q3	0.288	<.001	2.91	0.308	<.001	3.13	0.14	.03	2.75
Q1	0.274	<.001	2.93	0.398	<.001	3.08	0.12	.07	2.78

^a^MAE: mean absolute error.

Regardless of the relatively high correlations between the prediction set and the observable data, the best performing model did not predict GDT score values greater than 12, although the maximum attainable score in the questionnaire was 20 (Figure 1).

The reference point for the verification of H3 was the result of the correlation test-retest (*r*=0.713; A Cudo, personal communication via telephone, February 10, 2023), which turned out to be statistically significantly higher than the correlation between WBGD-4 and GDT (*z*=6.94, *P*<.001; [30]).

### Word Clouds

Word clouds were created for each WBGD-4 question response and translated into English using Google Translator (for details, see the Methods section). Figures S2-S5 in Multimedia Appendix 7 show the significant words that are indicative of the GDT scores for each question. Color codes indicate the *P* values, and the font size indicates the impact of the given word.

## Discussion

### Principal Findings

The main objective of this study was to investigate whether it is feasible to use question-based computational language assessment for the screening of gaming disorder as a supplement to state-of-the-art rating scales. Short open-ended responses analyzed through NLP offer a cost-effective way to deepen our understanding of gaming disorder without additional time investment. The results supported 2 out of the 3 preregistered hypotheses. The written responses analyzed using the model were correlated with the rating scale (H1). Moreover, incorporating more responses that reflect diverse perspectives on gaming further improved the model's performance (H2). However, there is still room for refinement, particularly in enhancing the questions to better align with the formal definition of gaming disorder (H3).

### Beyond the Rating Scales

Language-based assessments analyzed with transformer language models yield satisfactory results. According to H1, there were statistically significant positive correlations between the estimates made using the model and the traditional rating scale. The correlation coefficient obtained using ridge regression analysis (*r*=0.476) turned out to be statistically significant and moderate. We compared the performance of the model for women and men separately. The results turned out to be generally similar for both genders, with the male subsample showing slightly higher correlation coefficients for each combination of questions. However, the mean absolute errors were consistently lower for the female subgroup. The sample we studied was gender-balanced (with a slight predominance of women), so there is no reason to believe that a slightly worse model performance for women could result from an insufficient amount of data from women used to train the model. Taking into account the lower mean result of GDT and its standard deviation among women, it can be concluded that the lower MAE for women, despite weaker correlations, is due to the fact that the results were more uniform. In the future, it may be necessary to create separate models for women and men.

These results confirm that the analysis of the statements on the risk of gaming disorder may provide important information and potentially support more traditional screening methods. Taking into account the potential benefits of supplementing traditional methods, especially the possibility of precise determination of their mental states by the examined person and increased resistance to manipulation of the results, the collected results encourage further exploration of this direction.

### Optimizing the Accuracy

The questions we used to estimate the risk of gaming disorder were intended to reflect the questions a clinician would ask to estimate the risk of gaming disorder. Each of them touched on the problem of gaming disorder from a different perspective, and an experienced diagnostician may be able to synthesize the answers and assess the risk of gaming disorder. Comparing the prediction of the best GDT score from a single question (*r*=0.406, Question 4) with the score from the 4 questions (*r*=0.476) clearly demonstrates that applying more information from the questions about the same phenomenon but from different angles guarantees a significant improvement in predictive accuracy, which is in line with H2. It is worth noting that the combination of all 4 questions in comparison with only 3 questions (without Q1) worked similarly in the general sample. Notable differences in performance appeared only for the male subsample. Thus, in the future, one may consider reducing the number of questions in the WGBD-4 to 3, especially in samples dominated by women. This would result in a reduction in the time required to complete it by up to 25%. However, based on the data obtained, we recommend using the 4-item version, which may be more reliable.

Depending on the content of the questions, deeper and more comprehensive reflections of the gaming contexts may be revealed. This can be seen in our results, for example, in the different words used for different questions (Table 4 and Figures S3-S6 in Multimedia Appendix 7). There is room for improvement in this case, because in the reported study, we did not differentiate the response format, but it has been shown that such a procedure can also improve the validity of the criterion [6].

### Ecological Versus Criterion Validity Trade-Off

In H3, we predicted that the criterion validity of the trained model would approach the theoretical maximum limit as measured by the rating scale's test-retest reliability. At the time of the preregistration, this value was not known as the data collection was not completed; however, later results showed a test-retest correlation of *r*=0.713 for the Polish GDT (A Cudo, personal communication via telephone, February 10, 2023). This turned out to be a very high goal, but similar correlations are achievable for comparable models in other areas of mental well-being [6]. Our correlation coefficient was only *r*=0.476, which is moderate to strong in terms of conventional rules but statistically significantly lower than the reliability of the rating scale. Therefore, H3 was rejected. In other words, we achieved a satisfactory and promising result, but a comparison of the precision of our model’s predictions with the test-retest reliability of the current self-report tool shows that there is a lot of room for development. It is also worth noting that rating scales are only one of the ways of capturing mental phenomena; therefore, in the future, the point of reference for WBGD-4 should be more direct sources of information about gaming disorder. In retrospect, it can be said that H3, which we preregistered, was overly optimistic.

Subsequent studies can significantly increase the level of validity of the criterion by further improving the method or the validation criteria. Attention should be paid to the strategy of formulating open queries to encourage participants to write. In our study, we decided to prioritize ecological validity, that is, to ask questions in a way that would sound natural during an actual therapeutic encounter. Thus, we deliberately avoided formulating questions directly corresponding to items on the scale used as a comparative measure (GDT; [2]), which also allowed us to avoid criterion contamination [31]. As a result, in our questions and thus in the answers we received, there were aspects of gaming disorder that were not reflected in the rating scale (eg, regarding the motivations for gaming in Q3 of the WBGD-4). On the other hand, the rating scale directly asks questions about aspects of gaming disorder that we have not asked (eg, difficulty controlling playing time: Q3 from GDT). In fact, only Q4 from GDT and Q4 from WGBD-4 overlap to some extent. With this strategy in mind, a high correlation with rating scales should not be expected, but a high correlation with the diagnostician’s evaluation should. This issue requires further research. In subsequent studies, the model estimates based on the answers to the questions used in this study should be compared with the results of extensive diagnostics. However, it is worth emphasizing that the written statements of the respondents, even collected in this way, gave a high correlation score with the screening rating scales. In the future, adding prompts directing respondents' attention to the criteria proposed by WHO and that are reflected in the GDT should be considered. This should increase the numerical value reflecting the validity of the criterion without losing ecological validity.

### Opportunity to Bypass the Risk of Distorted Responses

A commonly known limitation of the reliability of psychological evaluation is the tendency of people to adapt their answers to the standards they perceive as expected. As we showed in the Introduction, simply supplementing self-descriptive data collection based on rating scales with text statements can contribute to a partial reduction of this problem. However, even greater promise is demonstrated by the possibility of analyzing behavior through the content of spontaneous statements by the participants. Research shows that it is possible to estimate mental states by processing samples of natural utterances on social networks [32]. Undoubtedly, spontaneous statements are different from answers to a direct prompt and, therefore, may be less informative. However, social networks that are detected suggest that identifying the risk of gaming disorder based on content posted on the internet may also be feasible.

### Limitations and Future Directions

This study focused on whether language-based risk assessments of gaming disorder can be used in the screening of this condition. However, the assumed reference point is not the grounded truth of this construct; it is a somewhat indirect measure. Thus, the first limitation is that, by showing even a strong correlation between language-based assessments and the traditional rating scale, we only indirectly proved that the former correspond to the condition of interest. This concern is shared with other developments in assessment tools where testing the validity of psychometric tools is often limited to comparing them with other previously validated tools. For example, Pontes et al [2] correlated their tool with the older scale (Internet Gaming Disorder Scale-Short-Form [33]) to verify the concurrent validity. In the future, the validity of the criterion should be on theoretically significant variables, such as behaviors or clinical diagnoses. This is especially important because WGBD-4 was optimized primarily to correspond to a clinical assessment and only secondary to the gaming disorder rating scales.

Second, as all subjects were Polish, it is important to note that the sample was not representative of other nationalities. We did not control the race of the participants. Taking into account the homogeneity of the Polish society, where the overwhelming majority are White people, the generalizability of the results is limited. Furthermore, the study was conducted in Polish, which is used almost exclusively by Polish citizens. In the future, WGBD-4 should be validated in other languages and cultural contexts due to the heterogeneity of the sample and the generalizability of our findings. Third, the study was conducted online. Even if the comfort associated with completing the survey without witnesses may have fostered the openness and truthfulness of some respondents’ answers, it may also be associated with less effort put into the answers by other respondents and partly explains the fact that we needed to remove almost one-fifth of the collected responses. From a practical standpoint, the effort put into verifying the correctness of responses or even correcting simple errors like typos could be reduced by collecting data under more controlled and mobilizing conditions. It is worth noting that decisions on how to collect data in the future should also depend on the objectives of a particular study. Data collected online may be more appropriate if the goal is to develop screening methods based on the analysis of text found online. On the other hand, if similar language models were to support the process of diagnosis or therapy, then data collected in person might be more useful.

Fourth, our study used HerBERT to create numerical embeddings for WBGD-4 responses; however, there are also other models available for the Polish language, such as Polish-GPT2-XL or BERT-base-polish-cased-v1. It could also be interesting to explore to what extent Polish-specific models outperform more universal models (eg, for the English language). Since the aim of our study was to explore the feasibility of using such tools to support the process of identifying people at risk of gaming disorder, we did not compare the performance of different models. However, such research would be advisable in the future.

Fifth, the distribution of gaming disorder in the population turned out to be strongly skewed, and the percentage of people with gaming disorder according to the GDT criterion (2.4%) was small, but similar results have been observed in previous studies (eg, [34]). This may have significantly lowered the validation measure in our case, and a study based on an equal number of people with gaming disorder and normal controls may have benefited the NLP training process. Due to the nature of the model training process, it is predisposed to give the most likely answer not only in the light of the content of the answers but also in terms of prior probability. In other words, in an environment where extreme scores are rare, it “does not pay off” for the model to indicate them. However, it is worth noting that a similar phenomenon would also occur if the data on which the model was trained came from numerical answers to the rating scale questions. To minimize this limitation, it is necessary to provide the model with more data from people with gaming disorder in the future to help it distinguish those at risk or impaired from healthy gamers.

### Conclusion

We showed that prompted short text responses can be used to assess the risk of gaming disorder. There is much room for improvement here, which can be achieved if the prompts correspond directly to the diagnostic guidelines and the content of the rating scales. Furthermore, increasing the amount of available data will have a beneficial effect on the reliability of the estimates. The presented results are a strong indication that, in the near future, language, the basic way of communicating mental states of people in natural situations, can be used to support the screening and diagnosis of gaming disorder. By leveraging NLP, researchers and clinicians can analyze vast amounts of unstructured text data from diverse sources such as social media, gaming forums, and clinical notes, uncovering nuanced patterns and sentiments associated with gaming behaviors. This innovative approach enables the identification of subtle linguistic markers that may indicate the presence of gaming disorder, offering a more comprehensive and dynamic understanding of the condition. We believe that using NLP can not only enhance the accuracy of diagnoses but also enable continuous, real-time monitoring and early detection, ultimately paving the way for more effective and timely interventions in the realm of gaming disorder. Any progress in this direction will contribute to the development of existing methods and the overcoming of their limitations. Analogous methods are already being developed in other fields of psychology, medicine, or neurosciences, and we anticipate that, in the near future, the use of NLP will improve the screening process, making it more multifaceted and natural for the benefit of diagnosticians and patients.

## Appendix

Multimedia Appendix 1 Polish version of the GDT scale, utilized in the study.

Multimedia Appendix 2 Polish version of the PHQ9 scale, utilized in the study.

Multimedia Appendix 3 Polish version of the GAD7 scale, utilized in the study.

Multimedia Appendix 4 Polish versions of the SWLS and HiLS scales, utilized in the study.

Multimedia Appendix 5 Polish version of the Gaming Involvement scale, utilized in the study.

Multimedia Appendix 6 GDT distribution chart.

Multimedia Appendix 7 Word clouds.

## Abbreviations

BERT: Bidirectional Encoder Representations from Transformers
GDPR: General Data Protection Regulation
GDT: Gaming Disorder Test
ICD: International Classification of Diseases
MAE: mean absolute error
NLP: natural language processing
PHQ-9: 9-item Patient Health Questionnaire
WBGD-4: Word-Based Gaming Disorder Test
WHO: World Health Organization

## Data Availability

The data, code, and materials [14], as well as the preregistration time-stamped for the study protocol [15], are available for download on the Open Science Framework (OSF). Data access is open after creating an OSF account.
