# Supplementary material for: Use of 4 Open-Ended Text Responses to Help Identify People at Risk of Gaming Disorder: Preregistered Development and Usability Study Using Natural Language Processing
Source: JMIR Serious Games. 2024 Dec 31;12:e56663. doi: 10.2196/56663 (PMC11733516; doi:10.2196/56663)
Supplement: Multimedia Appendix 1 [file games_v12i1e56663_app1.pdf]

The Polish Gaming Disorder Test (GDT) (Cudo et al., 2022)

Instrukcja: Poniższe pytania dotyczą Twojej aktywności jako gracza podczas ostatniego roku (tzn. w ciągu ostatnich 12 miesięcy). Aktywność gracza oznacza każdą aktywność związaną z grą odtwarzaną z komputera/laptopa, konsoli do gier lub innego urządzenia (np. telefonu komórkowego, tabletu itp.), zarówno online, jak i offline.

Proszę wskazać, jak często w ciągu ostatnich dwunastu miesięcy do dnia dzisiejszego średnio występowały następujące problemy.

	Nigdy	Rzadko	Czasami	Często	Bardzo często
1. Miałam/em trudności z kontrolowaniem mojej aktywności związanej z grami.	○	○	○	○	○
2. Nadawałam/em coraz większy priorytet graniu nad innymi zainteresowaniami życiowymi i codziennymi czynnościami.	○	○	○	○	○
3. Kontynuowałam/em granie mimo pojawienia się negatywnych konsekwencji.	○	○	○	○	○
4. Doświadczyłam/em poważnych problemów życiowych (np. osobistych, rodzinnych, społecznych, edukacyjnych, zawodowych) z powodu nasilenia moich zachowań związanych z graniem.	○	○	○	○	○

Interpretacja wyników:

Ostateczny wynik można uzyskać poprzez zsumowanie wszystkich czterech pozycji IGT. Wynik ten może znajdować się w przedziale od minimum 4 do maksimum 20 punktów, przy czym wyższe wyniki wskazują na wyższy stopień „Zaburzeń związanych z graniem”. W celu odróżnienia graczy z zaburzeniami od graczy bez zaburzeń, badacze powinni sprawdzić, czy uczestnicy spełnili wszystkie cztery kryteria diagnostyczne zawarte w poszczególnych pozycjach GDT, biorąc pod uwagę odpowiedzi "4: Często" lub "5: Bardzo często", co można interpretować jako potwierdzenie kryterium.
