# Supplementary material for: Use of 4 Open-Ended Text Responses to Help Identify People at Risk of Gaming Disorder: Preregistered Development and Usability Study Using Natural Language Processing
Source: JMIR Serious Games. 2024 Dec 31;12:e56663. doi: 10.2196/56663 (PMC11733516; doi:10.2196/56663)
Supplement: Multimedia Appendix 2 [file games_v12i1e56663_app2.pdf]

KWESTIONARIUSZ ZDROWIA PACJENTA-9 (PHQ-9)

**Jak często w ciągu ostatnich 2 tygodni dokuczały Panu/Pani następujące problemy?** *(Proszę zaznaczyć odpowiedź znakiem "✔")*	**Wcale nie doku-czały**	**Kilka dni**	**Więcej niż połowę dni**	**Niemal codzien-nie**
**1.** Niewielkie zainteresowanie lub odczuwanie przyjemności z wykonywania czynności	0	1	2	3
**2.** Uczucie smutku, przygnębienia lub beznadziejności	0	1	2	3
**3.** Kłopoty z zaśnięciem lub przerywany sen, albo zbyt długi sen	0	1	2	3
**4.** Uczucie zmęczenia lub brak energii	0	1	2	3
**5.** Brak apetytu lub przejadanie się	0	1	2	3
**6.** Poczucie niezadowolenia z siebie — lub uczucie, że jest się do niczego, albo że zawiódł/zawiodła Pan/Pani siebie lub rodzinę	0	1	2	3
**7.** Problemy ze skupieniem się na przykład przy czytaniu gazety lub oglądaniu telewizji	0	1	2	3
**8.** Poruszanie się lub mówienie tak wolno, że inni mogliby to zauważyć? Albo wręcz przeciwnie — niemożność usiedzenia w miejscu lub podenerwowanie powodujące ruchliwość znacznie większą niż zwykle	0	1	2	3
**9.** Myśli, że lepiej byłoby umrzeć, albo chęć zrobienia sobie jakiejś krzywdy	0	1	2	3

FOR OFFICE CODING ___0___ + ______ + ______ + ______

=Total Score: ______

Jeżeli zaznaczył/-a Pan/Pani którekolwiek z problemów, jak bardzo utrudniły one Panu/Pani wykonywanie pracy, zajmowanie się domem lub relacje z innymi ludźmi?

W ogóle nie utrudniły	Trochę utrudniły	Bardzo utrudniły	Niezmiernie utrudniły
☐	☐	☐	☐

Opracowanie: Dr Robert L. Spitzer, Dr Janet B.W. Williams, Dr Kurt Kroenke oraz współpracownicy z wykorzystaniem grantu oświatowego od firmy Pfizer Inc. Zgoda na powielanie, tłumaczenie, przedstawianie lub rozprowadzanie niniejszego dokumentu nie jest wymagana.
