# Supplementary material for: Use of 4 Open-Ended Text Responses to Help Identify People at Risk of Gaming Disorder: Preregistered Development and Usability Study Using Natural Language Processing
Source: JMIR Serious Games. 2024 Dec 31;12:e56663. doi: 10.2196/56663 (PMC11733516; doi:10.2196/56663)
Supplement: Multimedia Appendix 3 [file games_v12i1e56663_app3.pdf]

GAD-7

Jak często w ciągu ostatnich 2 tygodni dokuczały Panu(i) następujące problemy? *(Proszę zaznaczyć odpowiedź znakiem "✔")*	**Wcale nie dokuczały**	**Kilka dni**	**Więcej niż połowę dni**	**Niemal codziennie**
**1.** Czuł(a) się Pan(i) podenerwowany(a), niespokojny(a), mocno spięty(a)	0	1	2	3
**2.** Nie mógł(a) Pan(i) przestać się martwić albo zapanować nad tym	0	1	2	3
**3.** Za bardzo się Pan(i) martwił(a) różnymi rzeczami	0	1	2	3
**4.** Miał(a) Pan(i) trudności z relaksowaniem się	0	1	2	3
**5.** Był(a) Pan(i) tak niespokojny(a), że nie mógł(a) usiedzieć na miejscu	0	1	2	3
**6.** Łatwo stawał(a) się Pan(i) rozdrażniony(a) lub poirytowany(a)	0	1	2	3
**7.** Obawiał(a) się Pan(i), tak jakby miało się stać coś strasznego	0	1	2	3

(*For office coding: Total Score T____ = ____ + ____ + ____*)

Opracowanie: Dr Robert L. Spitzer, Dr Janet B.W. Williams, Dr Kurt Kroenke oraz współpracownicy, z wykorzystaniem grantu oświatowego od firmy Pfizer Inc. Zgoda na powielanie, tłumaczenie, przedstawianie lub rozprowadzanie niniejszego dokumentu nie jest wymagana.
