# Supplementary material for: Use of 4 Open-Ended Text Responses to Help Identify People at Risk of Gaming Disorder: Preregistered Development and Usability Study Using Natural Language Processing
Source: JMIR Serious Games. 2024 Dec 31;12:e56663. doi: 10.2196/56663 (PMC11733516; doi:10.2196/56663)
Supplement: Multimedia Appendix 4 [file games_v12i1e56663_app4.pdf]

Satisfaction with Life & Harmony in Life Scale

Poniżej znajduje się pięć stwierdzeń, z którymi możesz się zgodzić lub nie. Używając poniższej skali od 1 do 7, wskaż, w jakim stopniu zgadzasz się z każdym stwierdzeniem. Proszę o otwartość i szczerość podczas odpowiadania.

[7 –Zdecydowanie zgadzam się
6 –Zgadzam się
5 –Raczej zgadzam się
4 –Ani zgadzam się, ani nie zgadzam się
3 –Raczej nie zgadzam się
2 –Nie zgadzam się
1 –Zdecydowanie nie zgadzam się]

1. W większości aspektów moje życie jest bliskie mojego ideału.

2. Warunki mojego życia są doskonałe.

3. Jestem zadowolony ze swojego życia.

4. Mój styl życia pozwala mi pozostawać w harmonii.

5. Większość aspektów mojego życia pozostaje w równowadze.

6. Żyję w harmonii.

Polskie tłumaczenie (PS, 10.2022) na podstawie:

Kjell, O. (2019). Abbreviated Three-Item Versions of the Satisfaction with Life Scale and the Harmony in Life Scale Yield Improved Psychometric Properties.

Kjell, O. N., & Diener, E. (2021). Abbreviated three-item versions of the satisfaction with life scale and the harmony in life scale yield as strong psychometric properties as the original scales. *Journal of Personality Assessment*, *103*(2), 183-194.
