# Supplementary material for: Use of 4 Open-Ended Text Responses to Help Identify People at Risk of Gaming Disorder: Preregistered Development and Usability Study Using Natural Language Processing
Source: JMIR Serious Games. 2024 Dec 31;12:e56663. doi: 10.2196/56663 (PMC11733516; doi:10.2196/56663)
Supplement: Multimedia Appendix 5 [file games_v12i1e56663_app5.pdf]

Gaming Involvement – Skala zaangażowania w gry wideo

Zainteresowanie grami wideo u wielu osób nie ogranicza się wyłącznie do grania. Niektórzy z nas w związku z tym hobby robią też inne, powiązane rzeczy. Zastanów się przez chwilę i pomóż nam zrozumieć Twoje aktywności związane z graniem.

Ile MINUT dziennie spędzasz na następujących czynnościach?

UWAGA: Jeśli nie wykonujesz wcale danej czynności, wpisz "0" w polu odpowiedzi.

	W ciągu jednego dnia powszedniego	W ciągu jednego dnia weekendu
1. Granie w gry wideo		
2. Rozmyślanie nad grami wideo		
3. Czytanie lub oglądanie poradników, recenzji, teorii, dodatkowego lore związanego z grami wideo		
4. Oglądanie streamów i gameplayów gier wideo (w tym rozgrywki e-sportowe)		
5. Rozmawianie lub pisanie na temat gier wideo		
6. Rozważanie kupna dodatkowego contentu i materiałów kolekcjonerskich związanych z grami (skinki, gamepassy, waluta w grę, figurki, t-shirty z motywami gier)		

To cite: Strojny, P., Kiszka, P., Starosta, J., Szyszka, P. D., Starzec, S., Winiarska, A., ... & Zajas, A. (2023). It’s not just about how long you play. Indirect gaming involvement and genre preferences in predicting gaming disorder risk: evidence from preregistered studies. *Frontiers in Psychiatry,* 14, 1230774.
