# Supplementary figures and images for: Use of 4 Open-Ended Text Responses to Help Identify People at Risk of Gaming Disorder: Preregistered Development and Usability Study Using Natural Language Processing
Source: JMIR Serious Games. 2024 Dec 31;12:e56663. doi: 10.2196/56663 (PMC11733516; doi:10.2196/56663)

**Multimedia Appendix 1**

Figure S1. GDT score distribution

*
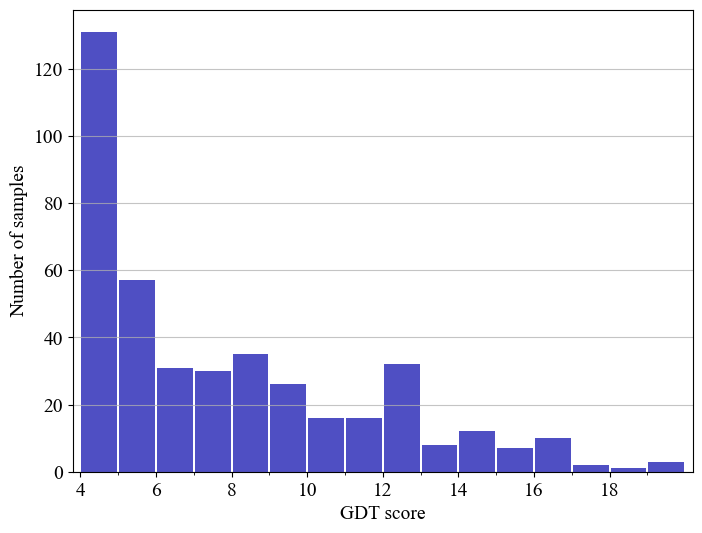
*

Supplement: Multimedia Appendix 6 [file games_v12i1e56663_app6.doc]
