# Supplementary material for: Use of 4 Open-Ended Text Responses to Help Identify People at Risk of Gaming Disorder: Preregistered Development and Usability Study Using Natural Language Processing
Source: JMIR Serious Games. 2024 Dec 31;12:e56663. doi: 10.2196/56663 (PMC11733516; doi:10.2196/56663)

The following Figures S2-S5 show the significant words that are indicative of the GDT scores for each question. Color codes for *P* values and font size for the impact of given word.

Figure S2. Question 1 word cloud. How does playing affect your life? The size of the word is proportional to its impact.


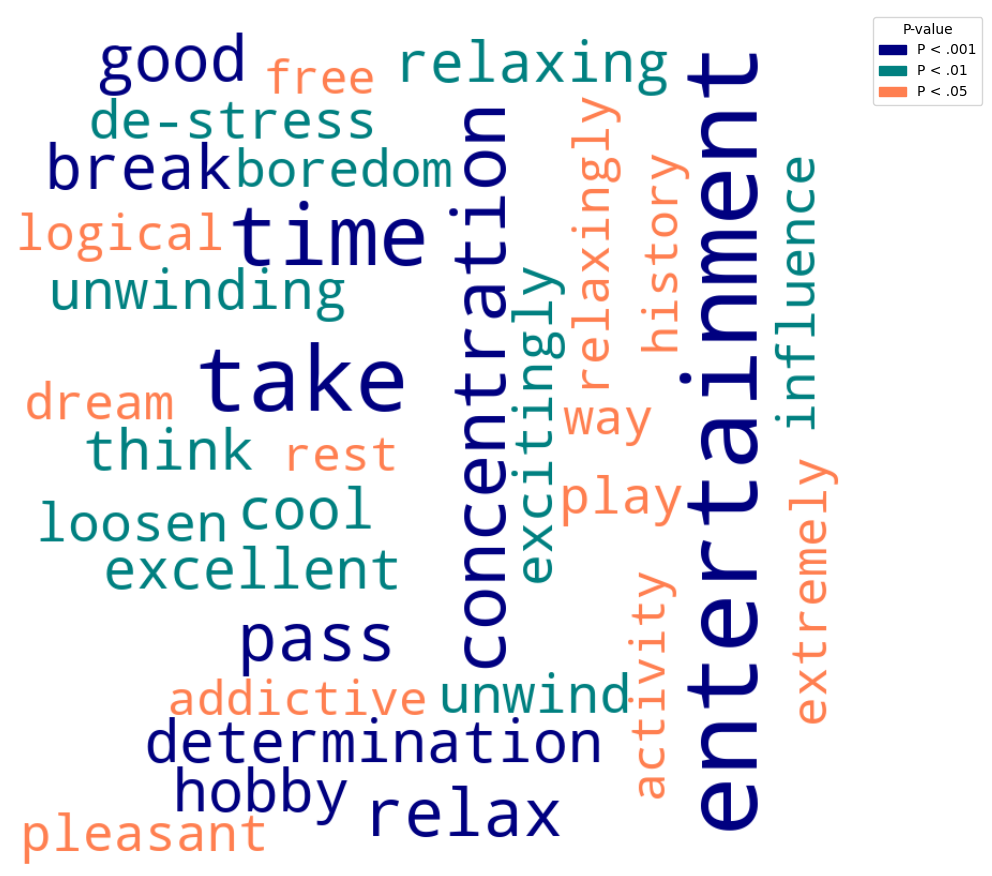


Figure S3. Question 2 word cloud. How does playing affect your emotions and thoughts when you are NOT playing? The size of the word is proportional to its impact.


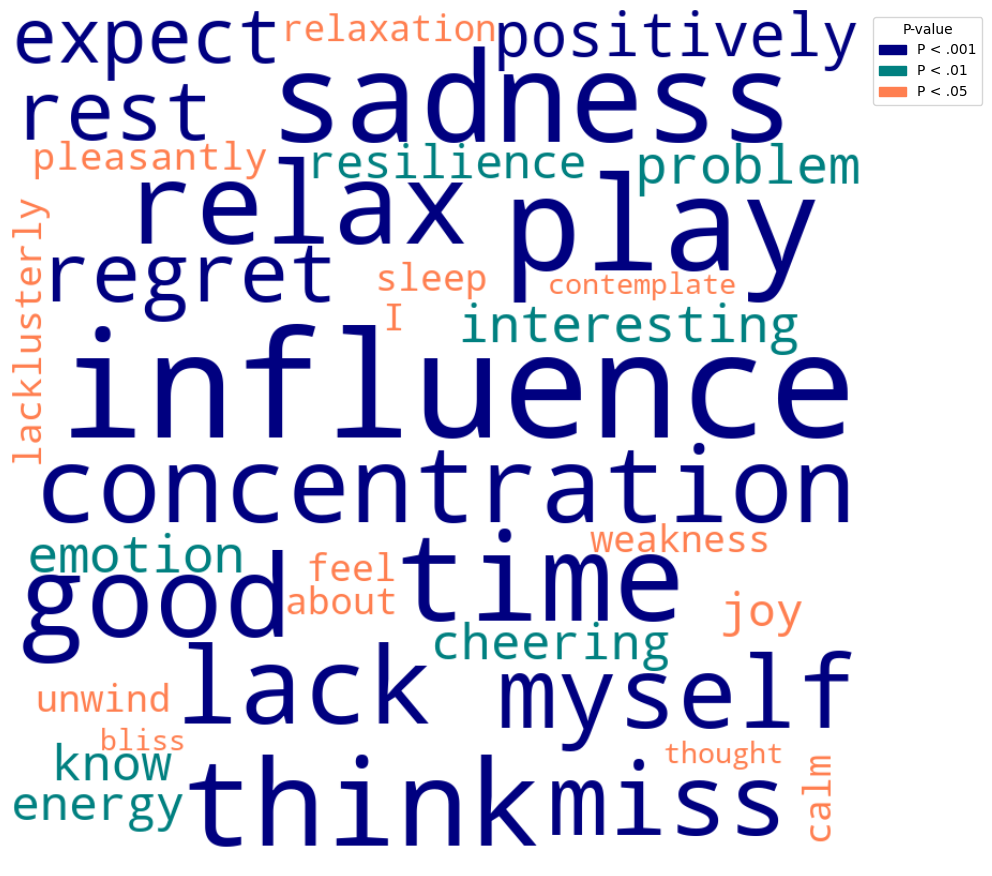


Figure S4. Question 3 word cloud. What needs do your activity related to games satisfy? The size of the word is proportional to its impact.


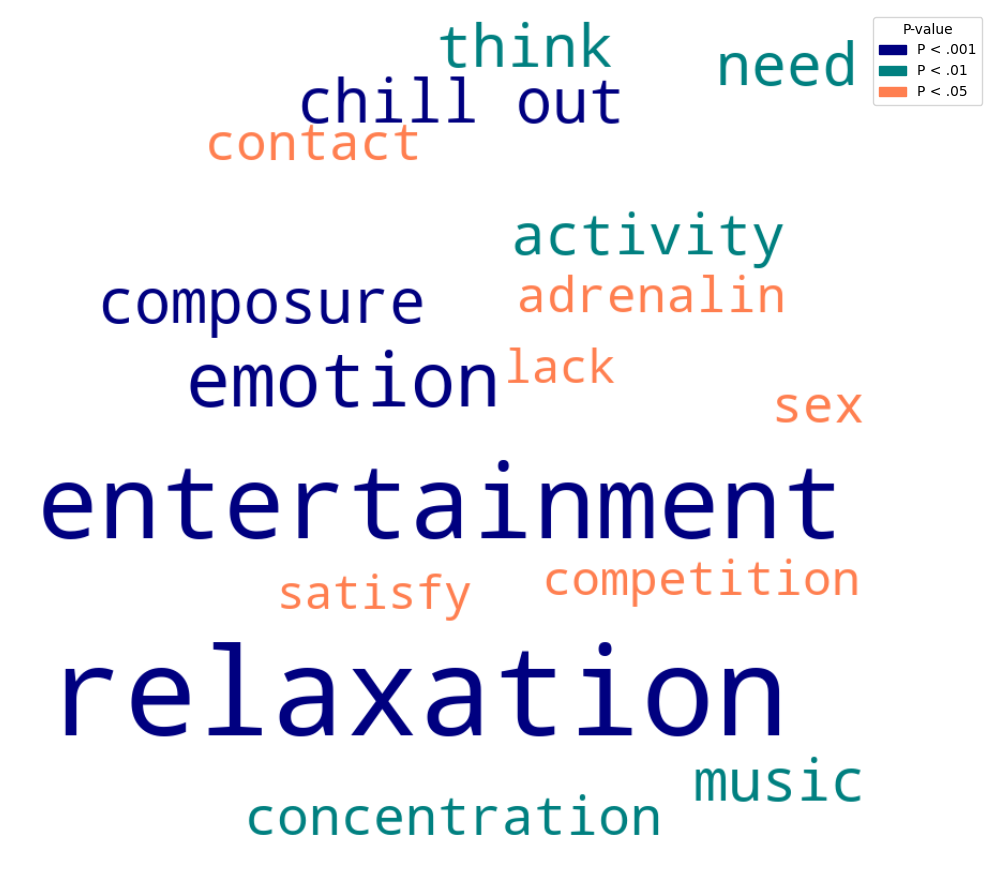


Figure S5. Question 4 word cloud. How do your loved ones react to your playing? The size of the word is proportional to its impact.


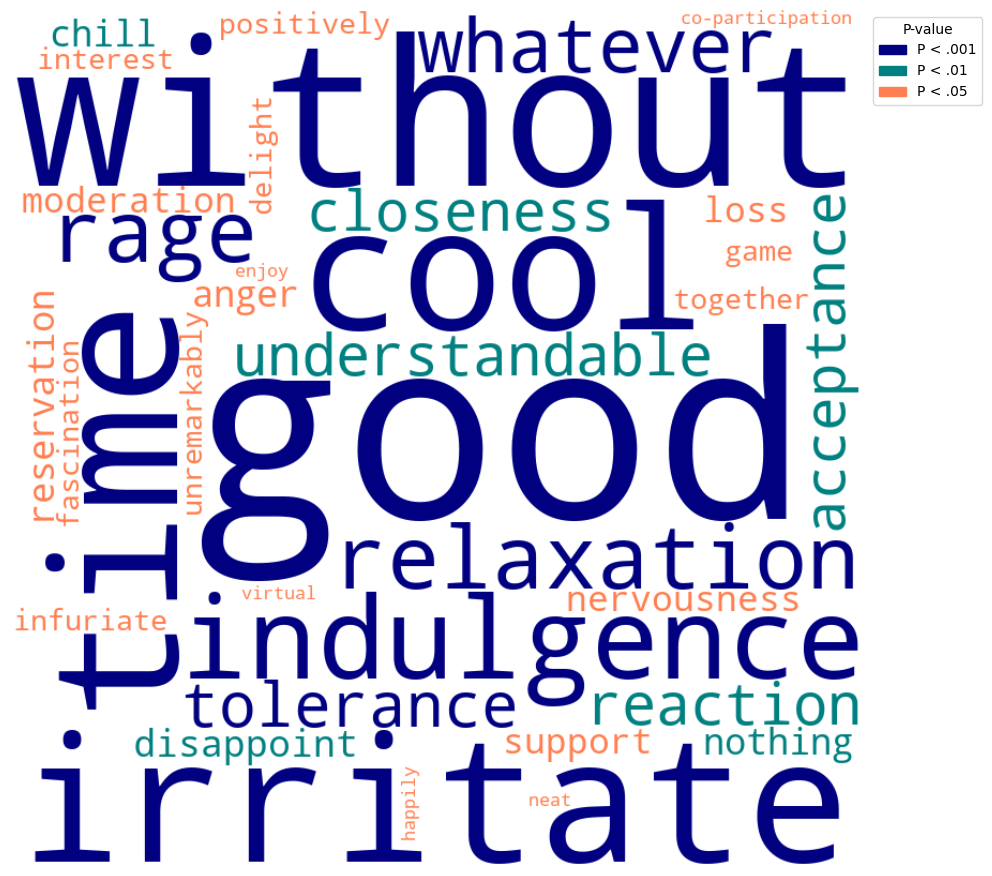

Supplement: Multimedia Appendix 7 [file games_v12i1e56663_app7.doc]
